# Supplementary material for: Temperature-Driven Divergence in Microbial Consortia and Physicochemical Functionality: A Comparative Study of High- and Medium-Temperature Daqu
Source: Microorganisms. 2025 Jun 5;13(6):1312. doi: 10.3390/microorganisms13061312 (PMC12195017; doi:10.3390/microorganisms13061312)
Supplement: Supplementary file 1 [file microorganisms-13-01312-s001.zip › microorganisms-3634095-supplementary.pdf]

Supporting Information for

Temperature-Driven Divergence in Microbial Consortia and Physicochemical Functionality: A Comparative Study of High- and Medium-Temperature *Daqu*

Figure captions

Figure S1. Experimental design of the study.

Table captions

Table S1. Changes in physicochemical indexes in medium-high temperature *Daqu* .

Table S2. Changes in enzyme activity in medium-high temperature *Daqu.*

Table S3. Alpha diversity of bacterial communities in medium-high temperature *Daqu.*

Table S4. Alpha diversity of fungal communities in medium-high temperature *Daqu.*

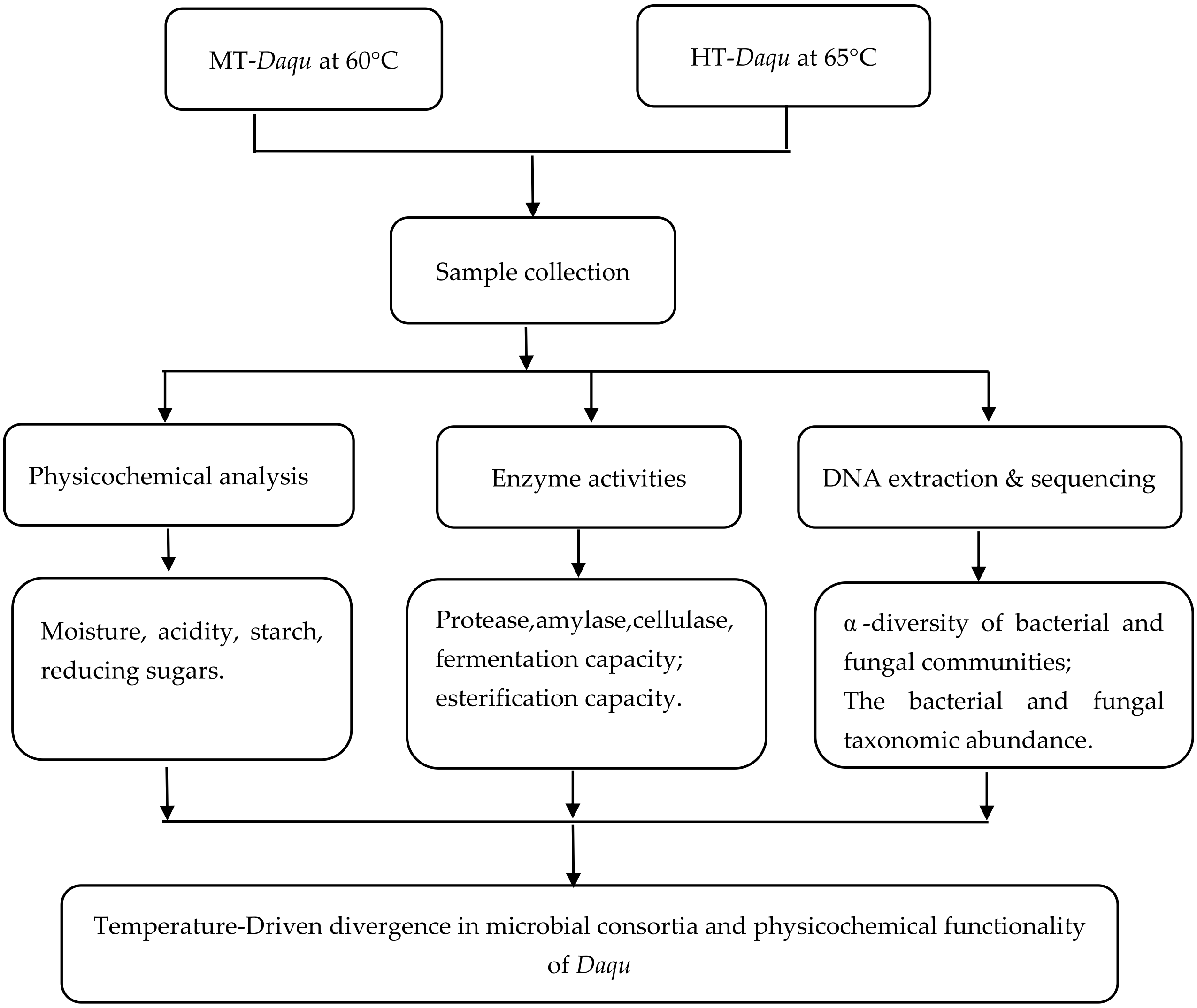

Figure S1 Experimental design of the study

Table S1. Changes in physicochemical indexes in medium-high temperature *Daqu*.

Parameter	HQ1	HQ2	HQ3	MQ1	MQ2	MQ3	*p*-value
Moisture (%)	8.88 ± 0.41^{a}	10.75 ± 0.21^{b}	10.30 ± 0.25^{b}	12.51 ± 0.42^{c}	11.68 ± 0.21^{d}	12.44 ± 0.23^{c}	<0.001
Acidity (mmol/10g)	1.77 ± 0.01^{a}	2.15 ± 0.01^{b}	1.70 ± 0.01^{c}	1.15 ± 0.12^{d}	1.23 ± 0.06^{d}	1.09 ± 0.02^{d}	<0.001
Reducing sugar (%)	0.87 ± 0.07^{a}	0.94 ± 0.08^{a}	0.75 ± 0.09^{b}	1.80 ± 0.10^{c}	1.86 ± 0.10^{c}	1.68 ± 0.18^{c}	<0.001
Starch (%)	55.74 ± 2.79^{a}	54.04 ± 0.92^{a}	57.93 ± 2.02^{b}	60.70 ± 1.54^{c}	61.20 ± 3.32^{c}	59.50 ± 2.84^{bc}	0.002

Notes: Values represent mean ± SD (n=3). Superscript letters denote significant differences (Tukey's HSD, $p < 0.05$). Groups sharing the same letter are not statistically different.

Table S2. Changes in enzyme activity in medium-high temperature *Daqu*.

Parameter	HQ1	HQ2	HQ3	MQ1	MQ2	MQ3	*p*-value
Neutral protease (μg/g·h)	17.71 ± 0.47^{a}	16.39 ± 0.49^{b}	14.39 ± 0.98^{c}	20.63 ± 0.55^{d}	23.74 ± 0.74^{e}	19.23 ± 0.89^{d}	<0.001
Acid protease (μg/g·h)	39.30 ± 0.91^{a}	51.68 ± 1.14^{b}	43.21 ± 1.33^{c}	16.64 ± 0.99^{d}	20.35 ± 1.13^{e}	27.54 ± 0.96^{f}	<0.001
Glucoamylase (mg/g·h)	133.73 ± 8.00^{a}	283.31 ± 3.45^{b}	256.25 ± 6.85^{c}	319.58 ± 8.61^{d}	515.47 ± 10.96^{e}	371.65 ± 18.60^{f}	<0.001
α-Amylase (g/g·h)	0.45 ± 0.01^{a}	0.40 ± 0.03^{b}	0.44 ± 0.03^{a}	0.59 ± 0.04^{c}	0.53 ± 0.02^{d}	0.70 ± 0.07^{e}	<0.001
Cellulase (mg/g·h)	4.05 ± 0.10^{a}	6.42 ± 0.12^{b}	4.44 ± 0.20^{c}	18.55 ± 0.52^{d}	21.27 ± 0.32^{e}	16.18 ± 0.54^{f}	<0.001
Hemicellulase (mg/g·h)	6.68 ± 0.20^{a}	5.16 ± 0.07^{b}	6.42 ± 0.06^{c}	13.45 ± 0.52^{d}	10.57 ± 0.54^{e}	14.19 ± 0.52^{f}	<0.001
Esterification [mg/(g·100h)]	15.69 ± 0.31^{a}	16.91 ± 0.64^{b}	14.59 ± 0.44^{c}	22.03 ± 1.14^{d}	22.69 ± 0.37^{d}	24.78 ± 0.56^{e}	<0.001
Fermentation [g/(g·72h)]	0.78 ± 0.02^{a}	0.36 ± 0.03^{b}	0.44 ± 0.01^{c}	1.08 ± 0.05^{d}	1.13 ± 0.07^{d}	1.24 ± 0.02^{e}	<0.001

Notes: Values represent mean ± SD (n=3). Superscript letters denote significant differences (Tukey's HSD, $p < 0.05$).

Table S3. Alpha diversity of bacterial communities in medium-high temperature *Daqu*.

Sample	Chao1Index					Simpson Index					Shannon Index				
	Replicate 1	Replicate 2	Replicate 3	Average	Average Absolute Deviation	Replicate 1	Replicate 2	Replicate 3	Average	Average Absolute Deviation	Replicate 1	Replicate 2	Replicate 3	Average	Average Absolute Deviation
HQ1	393.38	396.74	398.97	396.36	2.81	0.04	0.04	0.04	0.04	0.00	7.84	7.90	7.76	7.83	0.07
HQ2	427.34	427.53	427.68	427.52	0.17	0.03	0.03	0.03	0.03	0.00	4.81	4.82	4.80	4.81	0.01
HQ3	242.31	242.61	242.91	242.61	0.30	0.04	0.04	0.04	0.04	0.00	5.65	5.67	5.61	5.64	0.03
MQ1	642.03	642.05	642.01	642.03	0.02	0.98	0.98	0.98	0.98	0.00	4.34	4.36	4.36	4.35	0.01
MQ2	322.03	322.07	322.11	322.07	0.04	0.92	0.92	0.92	0.92	0.00	2.17	2.22	2.12	2.17	0.05
MQ3	577.11	577.12	577.13	577.12	0.01	0.94	0.94	0.94	0.94	0.00	1.80	1.72	1.76	1.76	0.04

Table S4. Alpha diversity of fungal communities in medium-high temperature *Daqu*.

	Chao1Index					Simpson Index					Shannon Index				
Sample	Replicate 1	Replicate 2	Replicate 3	Average	Average Absolute Deviation	Replicate 1	Replicate 2	Replicate 3	Average	Average Absolute Deviation	Replicate 1	Replicate 2	Replicate 3	Average	Average Absolute Deviation
HQ1	122.24	125.97	118.51	122.24	3.73	0.36	0.37	0.38	0.37	0.01	2.01	2.02	2.01	2.01	0.01
HQ2	42.69	47.83	52.97	47.83	5.14	0.63	0.63	0.63	0.63	0.00	1.53	1.58	1.48	1.53	0.05
HQ3	49.03	51.25	46.81	49.03	2.22	0.48	0.51	0.54	0.51	0.03	1.91	1.95	1.86	1.91	0.04
MQ1	209.79	209.79	209.79	209.79	0.00	0.99	0.99	0.99	0.99	0.00	8.41	8.44	8.46	8.44	0.02
MQ2	952.11	952.11	952.11	952.11	0.00	0.98	0.98	0.98	0.98	0.00	7.63	7.64	7.62	7.63	0.01
MQ3	636.34	636.34	636.34	636.34	0.00	0.64	0.64	0.64	0.64	0.00	3.72	3.72	3.72	3.72	0.00
